# Examining Reproductive Health Outcomes in Females Exposed to Polychlorinated Biphenyl and Polybrominated Biphenyl

**DOI:** 10.1038/s41598-020-60234-9

**Published:** 2020-02-24

**Authors:** Michael F. Neblett, Sarah W. Curtis, Sabrina A. Gerkowicz, Jessica B. Spencer, Metrecia L. Terrell, Victoria S. Jiang, M. Elizabeth Marder, Dana Boyd Barr, Michele Marcus, Alicia K. Smith

**Affiliations:** 1 Department of Gynecology and Obstetrics, Emory University School of Medicine, 101 Woodruff Circle NE, Ste 4300, Atlanta, GA 30322 Georgia; 2 Genetics and Molecular Biology Program, Laney Graduate School, Emory University School of Medicine, 101 Woodruff Circle NE, Ste 2205A, Atlanta, GA 30322 Georgia; 3 Department of Epidemiology, Emory University Rollins School of Public Health, 1518 Clifton Rd, Atlanta, GA 30322 Georgia; 4 Department of Environmental Health, Emory University Rollins School of Public Health, 1518 Clifton Rd, Atlanta, GA 30322 Georgia; 5 Department of Epidemiology, Rollins School of Public Health; Department of Environmental Health, Rollins School of Public Health; Department of Pediatrics, Emory University School of Medicine, 1518 Clifton Rd, Atlanta, GA 30322 Georgia; 6 Department of Gynecology and Obstetrics, Emory University School of Medicine, 101 Woodruff Circle NE, Ste 4217, Atlanta, GA 30322 Georgia

**Keywords:** Bioinformatics, Environmental impact, Environmental impact, Epidemiology

## Abstract

In 1973, accidental contamination of Michigan livestock with polybrominated biphenyls (PBBs) led to the establishment of a registry of exposed individuals that have been followed for > 40 years. Besides being exposed to PBBs, this cohort has also been exposed to polychlorinated biphenyls (PCBs), a structurally similar class of environmental pollutants, at levels similar to average US exposure. In this study, we examined the association between current serum PCB and PBB levels and various female reproductive health outcomes to build upon previous work and inconsistencies. Participation in this cross-sectional study required a blood draw and completion of a detailed health questionnaire. Analysis included only female participants who had participated between 2012 and 2015 (N = 254). Multivariate linear and logistic regression models were used to identify associations between serum PCB and PBB levels with each gynecological and infertility outcome. Additionally, a generalized estimating equation (GEE) model was used to evaluate each pregnancy and birth outcome in order to account for multiple pregnancies per woman. We controlled for age, body mass index, and total lipid levels in all analyses. A p-value of <0.05 was used for statistical significance. Among the women who reported ever being pregnant, there was a significant negative association with higher total PCB levels associating with fewer lifetime pregnancies ( *β* = −0.11, 95% CI = −0.21 to −0.005, p = 0.04). There were no correlations between serum PCB levels and the self-reported gynecological outcomes (pelvic inflammatory disease, endometriosis, polycystic ovarian syndrome, or uterine fibroids). No associations were identified between serum PCB levels and the prevalence of female infertility in women reporting ever having sexual intercourse with a male partner. There were no associations identified between serum PCB levels and pregnancy outcomes (singleton live births or miscarriages) or birth outcomes (preterm birth, birth weight, birth defects, hypertensive disorders of pregnancy, or gestational diabetes). PBB was not associated with any outcome. Further research is needed to determine if and how PCB may reduce pregnancy number.

## Introduction

Polychlorinated biphenyls (PCBs) and polybrominated biphenyls (PBBs) are synthetic chemicals that were once used as coolants in electrical equipment and as flame retardants in the manufacturing of plastics and electronics, respectively^1,2^. Although the production of PCBs ceased in 1977 and PBBs in 1976, concern over their health effects remains because of their continued persistence in the environment, the pervasiveness of their exposure in human populations, their lipophilicity and ability to accumulate in the food products, and their long biological half-life in the body^1–3^.

Continued exposure to both PBBs and PCBs is also a concern given that both PBBs and PCBs are endocrine-disrupting compounds (EDCs), meaning that they can interfere with the normal function of the endocrine system and may result in adverse health effects^1,2,4^. Multiple reproductive health and birth outcomes from exposure to PCBs and PBBs in women have been investigated, although results are not always consistent between experimental and epidemiological studies^4^. Specifically, women with greater PCB and PBB levels were both found to have effects on the menstrual cycle (shorter cycles with longer duration of bleeding)^5–7^, while a decrease in fecundability, possibly due to endocrine disruption in the oocyte, and longer time to pregnancy has been noted with high PCB levels^8–12^. Some studies have found associations with increased PCB and PBB exposure and spontaneous abortions^11,13^, while others have found no associations^3,14^. Several population-based studies have suggested PCB exposure may associate with increased risk of uterine fibroids^15,16^, polycystic ovarian syndrome (PCOS)^17^, and endometriosis^18^; however, PBB exposure has been less studied with no current associations identified with uterine fibroids or endometriosis^3,19^. Finally, studies of exposure to PCBs and PBBs have examined newborn Apgar scores^20^, birth weight^21–27^, preterm birth^21,23,24,28^, birth defects^29,30^, gestational diabetes^31–33^, and hypertensive disorders of pregnancy^34,35^ with inconsistent associations. The potential for adverse health effects from PBB and PCB exposure is also a continuing concern for future generations as studies have indicated there is transfer of both chemicals across the human placenta causing *in utero* exposure and high amounts of both chemicals in breastmilk causing early life exposure^36^.

Due to growing concerns of PBBs and PCBs as EDCs, the Endocrine Society released a second Scientific Statement urging researchers, physicians, and other healthcare providers to translate the science of endocrine disruption to improved public health interventions^4^. In order to further study the effects of these compounds, establishing long-standing exposure and health information will be invaluable. In 1973, over 6.5 million Michigan residents were exposed to PBB through accidental contamination of the food supply^37^. Later in subsequent generations, people were found to also be exposed via breast feeding and *in utero* transfer^38^. In an effort to understand the consequences of this exposure, the Michigan Department of Community Health initially enrolled over 5,000 individuals in a registry to monitor long- term health effects^39^. This cohort of exposed individuals and their children have now been followed for over 40 years. Additionally, although FireMaster did not contain PCBs, these Michigan families were also exposed to PCB from other sources, such as contaminated fish in the Great Lakes region and farms with PCB-lined silos^40^. This established geographic cohort of individuals in the Michigan PBB Registry provides an excellent opportunity to examine both PCB and PBB exposure and female reproductive health outcomes. This study will evaluate associations between reproductive outcomes and current serum PBB and PCB levels utilizing a larger sample size than some previous studies in this population.

## Methods

### Participant selection

This study utilized data collected as part of the Michigan PBB Registry. In 1976, the Michigan Department of Community Health (MDCH) began enrolling Michigan residents into a long-term registry of individuals believed to have the highest exposure to PBB after the agricultural accident. Included in the PBB Registry were residents living on quarantined farms, farm product consumers, and people who worked at the chemical plant that produced PBB and their families. Original recruitment of these participants has been described elsewhere in more detail^39^. Children of the original registry participants and other members of the community are currently being enrolled (http://pbbregistry.emory.edu/).

As part of continued effort to enroll members and inform the community of research findings, members of the PBB registry were mailed invitations to attend community meetings held throughout the state where recruitment was conducted between 2012 and 2015. Community meetings were also advertised in the local press; thus, individuals from the original registry as well as others who had lived in the state of Michigan during the time of contamination (1973–1974) or were offspring of those who lived in the state during that time^41^ attended and subsequently participated. For this study, participants had to be women between the ages of 18 and 59, had to have completed a detailed reproductive health questionnaire, and had to have a blood draw with recent PCB and PBB concentrations. Blood draw for PBB and PCB assessment and completing the health questionnaire occurred at one time point. A majority of the women in this study did not have a PBB or PCB exposure assessment at the time of their enrollment in the Michigan PBB Registry in the 1970’s. A total of 262 women met these criteria. Informed consent was obtained from each individual before participation. Study protocols were approved by the Institutional Review Board at Emory University, and all methods were performed in accordance to guidelines and regulations.

All demographic information was extracted from the health questionnaire and used to characterize the study population. Demographic variable believed to be relevant included: age, height and weight to calculate body mass index (BMI), race, income, insurance status, menopause status (>12 months with no period, blood test, or surgical removal of ovaries), smoking status, and serum levels of PCB, PBB, and total lipids. When demographic information was missing or unknown, these values were omitted while computing the mean, standard deviation, and range.

### Reproductive health assessment

Participants completed a detailed questionnaire which included questions on reproductive health. For each self-reported gynecological outcome evaluated (pelvic inflammatory disease, endometriosis, polycystic ovarian syndrome, or uterine fibroids), women were classified as having these conditions if the patient reported that the condition had been diagnosed by a doctor. Next, questions regarding infertility were evaluated only in women who reported ever having sexual intercourse with a male partner. Questions regarding infertility included: 1) any report of a six or twelve month time period in life with regular unprotected intercourse without becoming pregnant, 2) any report of a six or twelve month time period with regular unprotected intercourse and not achieving pregnancy, 3) any report of a problem or concern about ability to get pregnant, 4) a history of visiting a health care provider due to difficulty getting pregnant, and 5) any report of receiving fertility treatment. For each woman who reported a pregnancy, the number of pregnancies were evaluated. Additionally, for each pregnancy a woman reported, birth outcome (singleton live birth or miscarriage) was analyzed. For each singleton live birth, delivery outcomes (preterm birth [<37 weeks gestational age], birth weight in grams, self-reported low [<2500 g] or high [>4000 g] birth weight, report of any birth defects, report of hypertensive disorders of pregnancy, and report of gestational diabetes) were evaluated.

### Exposure assessment

There are 209 possible congeners of PCB and PBB that exist based on the position and number of a chlorine or bromine atoms around the biphenyl rings^42^. PBB-153 (2,2′4,4′5,5′ hexa-bromobiphenyl) constitutes approximately 61% of FireMaster FF-1, the commercial product that contaminated the food supply in the 1970s^43^. Exposure to four most biologically relevant congeners of PCB (118, 138, 153, and 180) and four congeners of PBB (77, 101, 153, 180) were previously analyzed in members of this registry using gas chromatography- tandem mass spectrometry^44^. The limit of detection (LOD) was 1.4 pg/mL for PCB-118, 1.2 pg/mL for PCB-138, 1.6 pg/mL for PCB-153, 0.7 pg/mL for PCB-180, 2 pg/mL for PBB-153, 4.5 pg/mL for PBB-77, 3.9 pg/mL for PBB-101, and 5.6 pg/mL for PBB-180. The extraction recovery ranged from 83.2–99.2%. The accuracy ranged from 89–119%, and the precision ranged from 2.8–8.5%. The value for any congener below the LOD in a sample was imputed as the LOD divided by the square root of 2^45^. Previous studies in this population have reported high correlation between PBB congeners, high correlation between PCB congeners, and low correlation between PBB and PCB congeners^46^. The congeners were summed by wet-weight concentrations to give total PCB and total PBB values per person (total PCB = PCB-118+ PCB-138+ PCB-153+ PCB-180; total PBB = PBB-77+ PBB-101+ PBB-153+ PBB-180). Eight samples failed quality control checks for their PCB-118 measurements due to unstable retention times and were excluded from further analyses (N = 254).

### Serum lipid measurement

As previously described^41,47^, a Triglyceride Quantification Assay Kit (Abnova Corporation) was used to measure the total triglyceride content in serum, and a Cholesterol Assay Kit (Caymen Chemical Company) was used to measure total cholesterol content in serum. Both were done according to manufacturer’s instructions. Total lipid content was calculated based on methods previously described and was used as a covariate in our analysis^48^.

### Statistical analysis

Total PCB and PBB values were natural log-transformed for all statistical analyses to reduce the influence of extreme outliers. Multivariate logistic regression models were used to identify correlations between serum PCB and PBB levels and the gynecological health outcomes (N = 254). Multivariate logistic regression models were also used to identify associations between serum PCB and PBB levels and questions regarding female infertility in women reporting ever having sexual intercourse with a male partner (N = 234). Next, of the women who reported ever being pregnant (N = 192), we analyzed for an association between serum PCB and PBB levels and number of pregnancies using a Poisson regression. Age, BMI, and total lipid levels were covariates in all analyses. We controlled for PBB in all PCB analyses and PCB in all PBB analyses. Sensitivity analyses were conducted without controlling for total lipids levels in all of the models to test whether that altered the association. Sensitivity analyses were also performed on questions regarding infertility and number of pregnancies by including the presence of any additional infertility risk factors (presence of endometriosis, polycystic ovarian syndrome, uterine fibroids, or sexually transmitted infections with a link to infertility) to the models as a covariate with age, BMI, and total lipid levels. Sexually transmitted infections with a strong link to infertility that were asked in this questionnaire included gonorrhea, chlamydia, and trichomonas.

A generalized estimating equation (GEE) model using an exchangeable covariance structure was used to evaluate pregnancy and singleton live birth (SLB) outcomes, in order to control for multiple pregnancies per women. Pregnancies that ended in abortion, multiple gestation, stillbirth, or were ectopic were excluded in these analyses due to small sample size (N = 28). Current pregnancies were also excluded (N = 6). Of the pregnancy outcomes (N = 449), singleton live births and miscarriages were then evaluated with the transformed serum PCB and PBB levels controlling for age, BMI, and total lipid levels in both analyses. A sensitivity analysis was then performed without lipid levels as a covariate. A sensitivity analysis was also performed for the miscarriage outcome by including the presence of any additional risk factors (polycystic ovarian syndrome, uterine fibroids, hypertensive disorders of pregnancy, and diabetes in pregnancy) to the models as a covariate with age, BMI, and total lipid levels. For all analyses, a logistic regression model was used in the generalized estimating equation.

The remaining delivery outcomes were examined from the singleton live birth outcomes (N = 369). PCB and PBB levels were evaluated in relation to preterm birth, low birth weight, high birth weight, birth weight (in grams), birth defects, hypertensive disorders of pregnancy (gestational hypertension, preeclampsia, or eclampsia), and gestational diabetes again controlling for age, body mass index, and total lipid levels in all analyses. A sensitivity analysis was then performed without lipid levels as a covariate. For preterm birth outcomes, a sensitivity analysis was performed including the presence of any additional preterm birth risk factors (polycystic ovarian syndrome, uterine fibroids, hypertensive disorders of pregnancy, and diabetes in pregnancy) to the models as a covariate with age, BMI, and total lipid levels. Lastly, a sensitivity analyses was likewise performed for low birth weight, high birth weight, and birth weight (in grams) including the presence of any additional birth weight risk factors (hypertensive disorders of pregnancy and diabetes in pregnancy) to the models as a covariate with age, BMI, and total lipid levels. For all analyses using dichotomized variables, a logistic model was used in the generalized estimating equation, for analyses using a continuous variable (birth weight in grams), a linear model was used in the generalized estimating equation.

All statistical analyses were performed using the R statistical package version 3.5.1. An alpha value of 0.05 was used to determine statistical significance.

## Results

A total of 254 women met inclusion criteria for this study. The participant demographic characteristics are shown in Table 1. Among them, the average age at time of sample collection was 41 years and ranged from 18 to 59 years. The study population was 95% White/Non-Hispanic. The average BMI was 29.2, with 68% of participants being classified as at least overweight (BMI > 25). Ninety percent had health insurance to see a medical provider, although only 7% reported that their insurance covered fertility treatments (fully or partially). Additionally, 23% had a household income less than $20,000, which could provide difficulty with affording specialty care (i.e. fertility services) either with or without health insurance. Total serum PCB levels ranged from 0.03 to 2.60 ng/mL (geometric mean 0.43 ng/mL) while serum PBB levels ranged from 0.01 to 4.96 ng/mL (geometric mean 0.10 ng/mL). Total PCB and total PBB were moderately correlated in this study population (r = 0.39; p = 1.03e-10). The number of self-reported pregnancies for each woman in the cohort ranged from zero to ten. There were no statistically significant correlations between total serum PCB or PBB levels and the self-reported gynecological outcomes (Table 2).

**Table 1:** Characteristics of women from the Michigan PBB Registry study of reproductive function (N = 254).

	Mean ± S.D.	Range
Age^**a**^, years at blood draw	40.94 ± 10.79	18.50–58.70
BMI (kg/m^2^)^**a**^	29.18 ± 7.59	17.38–58.58
Total PCB (ng/mL or ppb)^**b**^	0.43 ± 2.65	0.03–2.60
Total PCB (ng/g lipids)^**b**^	61.44 ± 2.68	4.46–517.48
Total PBB (ng/mL or ppb)^**b**^	0.10 ± 4.50	0.01–4.96
Total PBB (ng/g lipids)^**b**^	14.72 ± 4.47	0.87–823.10
Total Lipids (ng/g lipid)^**a**^	730.5 ± 215.5	336.4–1518.8
Number of pregnancies^**a**^	2.15 ± 1.74	0–10
**Race [n (%)]**		
White/Non-Hispanic	241 (94.8%)	
White/Hispanic	5 (1.9%)	
Hispanic	1 (0.3%)	
Native American	3 (1.1%)	
**Household Income [n (%)]**		
<$20 K	58 (22.8%)	
$20–50 K	56 (22.0%)	
$50–100 K	100 (39.3%)	
>$100 K	33 (13.3%)	
**Insurance [n (%)]**		
Yes	229 (90.2%)	
No	25 (9.8%)	
**Menopause [n (%)]**		
Yes	56 (22.1%)	
No	198 (77.9%)	
**Ever Smoked [n (%)]**		
Yes	86 (33.9%)	
No	168 (66.1%)	

^a^Arithmetic mean.

^b^Geometric mean.

**Table 2:** Associations between exposures and gynecological outcomes.

	N (%)	PCB		PBB	
		OR (95% CI)	P-value	OR (95% CI)	P-value
PID^a^	13 (5.1%)	1.36 (0.70 to 2.82)	0.37	1.04 (0.59 to 1.80)	0.88
Endometriosis	44 (17.3%)	1.02 (0.68 to 1.53)	0.92	1.04 (0.75 to 1.43)	0.78
PCOS^b^	23 (9.0%)	0.94 (0.56 to 1.57)	0.82	1.01 (0.64 to 1.57)	0.93
Uterine Fibroids	20 (7.8%)	0.71 (0.37 to 1.32)	0.29	0.91 (0.56 to 1.43)	0.69

Adjusted for age, BMI, and total lipid levels. Total PCB levels adjusted for total PBB and total PBB levels adjusted for total PCB. OR = odds ratio. CI = confidence interval.

^a^Pelvic inflammatory disease.

^b^Polycystic ovarian syndrome.

No statistically significant associations were identified between serum PCB or PBB levels and the prevalence of female infertility in women reporting ever having sexual intercourse with a male partner (Table 3). Among the women who reported ever being pregnant, there was a significant negative association, with higher total serum PCB levels associating with fewer lifetime pregnancies ($\beta$= −0.11, 95% confidence interval [CI] = −0.21 to −0.005, p = 0.04) shown in Table 4. No associations were found between PBB levels and number of pregnancies. Sensitivity analyses subsequently performed on questions regarding infertility and number of pregnancies were completed controlling for infertility risk factors, and the above findings remained consistent (Table S1). Total serum PCB levels continued to have a significant negative association with fewer lifetime pregnancies ($\beta$= −0.11, 95% CI = −0.21 to −0.005, p = 0.03).

**Table 3:** Association between exposures and infertility outcomes.

	N (%)	PCB		PBB	
		OR (95% CI)	P-value	OR (95% CI)	P-value
Any report of going 6+ months with regular unprotected intercourse and DID NOT achieve pregnancy?	44 (18.8%)	1.39 (0.95 to 2.05)	0.08	0.98 (0.69 to 1.36)	0.91
Any report of going 12+ months with regular unprotected intercourse and DID NOT achieve pregnancy?	26 (11.1%)	1.23 (0.77 to 1.99)	0.38	1.09 (0.71 to 1.64)	0.68
Any report of going 6+ months with regular unprotected intercourse in life without becoming pregnant?	119 (50.8%)	1.22 (0.91 to 1.66)	0.18	0.90 (0.69 to 1.16)	0.42
Any report of going 12+ months with regular unprotected intercourse in life without becoming pregnant?	77 (32.9%)	1.03 (0.75 to 1.43)	0.83	0.88 (0.67 to 1.15)	0.37
Have you ever had a problem or been concerned about possible problem with ability to get pregnant?	70 (29.9%)	0.93 (0.67 to 1.30)	0.68	1.04 (0.79 to 1.36)	0.77
Have you ever visited a health care provider, doctor, or clinic because you were having difficulty getting pregnant?	43 (18.3%)	0.79 (0.53 to 1.18)	0.26	0.92 (0.65 to 1.27)	0.62
Reports receiving fertility treatments	29 (12.3%)	0.86 (0.53 to 1.36)	0.52	0.83 (0.55 to 1.21)	0.36

Adjusted for age, BMI, and total lipid levels. Total PCB levels adjusted for total PBB and total PBB levels adjusted for total PCB. OR = odds ratio. CI = confidence interval.

**Table 4:** Associations between exposures and pregnancy outcomes.

	Mean (range)	PCB		PBB	
		${\boldsymbol{\beta }}$ (95% CI)	P-value	${\boldsymbol{\beta }}$ (95% CI)	P-value
**Number of pregnancies**	2.84 (0–10)	**−0**.**11 (−0**.**21 to −0**.**005)**	**0**.**04**	0.02 (−0.06 to 0.09)	0.64
**Pregnancy Outcomes**	N (%)	OR (95% CI)	P-value	OR (95% CI)	P-value
Singleton live birth (SLB)	369 (82.2%)	0.89 (0.65 to 1.22)	0.47	1.03 (0.83 to 1.26)	0.78
Miscarriage	80 (17.8%)	1.12 (0.82 to 1.52)	0.47	0.97 (0.79 to 1.19)	0.78
**SLB Outcomes**	**N (%)**	**OR (95% CI)**	**P-value**	**OR (95% CI)**	**P-value**
Preterm birth^a^	35 (9.5%)	0.98 (0.62 to 1.53)	0.93	0.99 (0.73 to 1.34)	0.97
Low birth weight^b^	23 (6.2%)	1.03 (0.62 to 1.70)	0.91	1.11 (0.77 to 1.61)	0.56
High birth weight^c^	39 (10.6%)	0.69 (0.43 to 1.12)	0.13	0.96 (0.70 to 1.30)	0.79
Birth defects	26 (7.0%)	0.93 (0.55 to 1.55)	0.78	0.86 (0.61 to 1.22)	0.40
Hypertensive disorders of pregnancy^d^	29 (7.9%)	1.49 (0.87 to 2.53)	0.14	1.29 (0.83 to 2.02)	0.25
Gestational diabetes	25 (6.8%)	1.06 (0.59to 1.87)	0.85	0.96 (0.56 to 1.67)	0.90
	**Mean (range)**	${\boldsymbol{\beta }}$ **(95% CI)**	**P-value**	${\boldsymbol{\beta }}$ **(95% CI)**	**P-value**
**Birth weight (grams)**	3351 g (1219–5415 g)	−65.2 (−160.5 to 30.1)	0.18	30.9 (−30.0 to 91.9)	0.32

Adjusted for age (current age & age at pregnancy), BMI, and total lipid levels. Total PCB levels adjusted for total PBB and total PBB levels adjusted for total PCB. OR = odds ratio. CI = confidence interval.

Bold indicates statistically significant values with p-value of <0.05.

^a^ < 37 weeks gestational age.

^b^Self-reported birth weight <2500 g.

^c^Self-reported birth weight >4000 g.

^d^Gestational hypertension, preeclampsia, or eclampsia.

Pregnancy outcomes, singelton live birth outcomes, and birth weight were then evaluted using a GEE model (Table 4). Of the pregnancy outcomes, the number of SLBs or miscarriages were not affected by total serum PCB or PBB levels. Finally, of the singleton live birth outcomes, there were no associations between total serum PCB or PBB levels and preterm births, self-reported low or high birth weight, birth defects, hypertensive disorders of pregnancy, gestational diabetes, or total birth weight. Sensitivity analyses examining miscarriage, preterm birth, and birth weight risk factors were performed, and the above findings remained consistent (Table S1).

Finally, when all analyses were performed without controlling for total lipid levels, the above conclusions remained consistent (Tables S2–S4). PCB exposure again continued to have a significant negative assocation with fewer lifetime pregnancies ($\beta \,$=  −0.11, 95% CI = −0.21 to −0.003, p = 0.04).

## Discussion

This cross-sectional study utilized data from 254 women in a registry of Michigan residents who were exposed to PBB from an agricultural accident over 40 years ago or were offspring of Michigan residents. Participants from this geographic and established cohort additionally had serum PCB and PBB levels measured which provides an excellent opportunity to examine PCB and PBB exposure and reproductive health outcomes. Compared with National Health and Nutrition Examination Survey (NHANES) data from 2003–2004, our study population had comparable PCB levels to the rest of the U.S. and had higher PBB exposure^49^, with 90.0% of women having more than the geometric mean level present in the general U.S. population^50^. Additionally, average current serum PBB levels of these women were lower than their PCB levels, likely due to continuing environmental exposure to PCBs.

Our findings did not indicate any associations between serum PCB or PBB exposure and any of the self-reported gynecological outcomes. Several studies have proposed that higher PCB exposure may increase the risk of developing endometriosis, while some epidemiological studies have failed to find any associatons^18,19^. An earlier study of the Michigan PBB cohort did not support an association between PBB exposure and endometriosis^19^. Another study looking at a diagnosis of uterine fibroids was not linked with serum PCB levels; however, an association was seen using PCB levels measured in omental fat^16^. Although we did control for lipids in our study, this may suggest measuring long-term exposure in fat with on-going PCB exposure may more accurately predict subsequent health outcomes. There are no current associations reported in the limited number of studies with PBB exposure and uterine fibroids^3^. Likewise, no relationships were identified between PCB exposure and incidence of PCOS, as suggested in a previous study^17^. This lack of a relationship in our study could be due to the etiology of PCOS, which is thought to be multifactoral, or that we may have been underpowered to detect an assocation.

We did not identify any association between serum PCB or PBB and female infertility, as has been suggested by other studies^8–10^. However, among women who reported ever being pregnant, total serum PCB levels correlated with fewer numbers of lifetime pregnancies, consistent with previous studies^8–12^. Finding an association between pregnancy number and PCB, but not infertility and PCB, could be due to recall bias (for infertility variables defined by months of unprotected sexual intercourse) or due to limited access to infertility care due to cost and/or insurance coverage (for infertility variables defined by seeing a provider or receiving fertility treatments). Additionally, it should be noted most women did not answer questions related to their intention to conceive, and thus we were not able to adjust for this potential confounder. Lower fecundity with PCB exposure was previously observed in the Longitudinal Investigation of Fertility and the Environment (LIFE) study in both females and males^51^. The mechanism in which PCB may decrease fecundity is not well understood. Previous studies have suggested hormonal disruption (production, transport, and/or elimination), as well as ovarian dysfunction^12,51^. Furthermore, other endocrine organs with a role in reproduction, such as the thyroid, have been shown to be affected by PHBs^41^.

Finally, our results add to the existing body of literature regarding PCB and PBB exposure and various pregnancy and birth outcomes. We did not find an association between increasing PCB or PBB levels and odds of miscarriage or live birth. Although an older study did find a significant association with PCB exposure and spontaneous abortions^13^, more recent studies with larger sample sizes did not support these findings^3,14^. There is evidence, however, that *in utero* exposure to PBB does increase the odds of a spontaneous abortion, suggesting that timing of exposure could alter health outcomes associated with exposure^11^. Our study did not evaluate whether age at exposure altered any of the associations between PBB and reproductive health outcomes as we did not have a large enough sample size with maternal PCB/PBB levels. Therefore, future studies should evaluate whether women who were exposed to PBB or PCB *in utero* have health effects not seen in the women exposed later in life, as has been reported by previous studies^3,11,30,52,53^.

Additionally, we did not find any evidence of increasing serum PCB or PBB levels and birth outcomes. Similar to our study, several previous studies have found no significant findings between PCB and PBB levels and preterm birth or birth defects^21,23,24,28,29^. A recent study found a negative relationship between PCB-153 exposure and birth weight^27^, but our study and older studies, have not identified an association with birth weight^21–24^. Some of these inconsistent results may be due to differences in design, exposure measurement, and background populations^27,54,55^. Younger age of exposure to PBB was strongly associated with increased birth weight among offspring; however, this association with maternal age at exposure has been suggested to potentially be an artifact^21,25,26^. Furthermore, very few studies have examined the interaction between PCB and PBB exposure and the development of gestational diabetes (GDM)^56^, although our results are consistent with the previous studies that have been done^31–33^. Hypertension has been shown to associate with PCB^57,58^, but very few studies have looked for associations with PBB or hypertensive disorders of pregnancy (gestational hypertension, pre-eclampsia, and eclampsia). A small Iranian study (N = 45) showed an increased risk with PCB and pre-eclampsia^34^ while our study and a larger cohort study did not find any increased risks of developing any hypertensive disorders of pregnancy^35^.

This study benefited from several strengths. We used human biological serum samples for measuring PCB/PBB exposure as compared to other studies which used more indirect measures of exposure, such as fish consumption or zip codes. Additionally, the women’s serum PCB levels in our study population were comparable to national geometric mean levels based on 2003–2004ES data when evaluating for all associations, suggesting that this is a reasonable population to model exposure-related health outcomes in the general U.S. population^59–61^. Each participant took time to complete a comprehensive detailed questionnaire with many reproductive heath questions allowing us to study multiple outcomes of interest. Furthermore, to the best of our knowledge, this is the first paper to look for assocations between PBB and many of the detailed female health outcomes, such as PCOS. Finally, this study utilized a larger sample size compared to previous epidemiological studies of PBB exposure.

Our study was also subject to several limitations. First, there is potential bias because the responses to the detailed health questionnaire rely on self-report rather than medical records. Additionally, because this cohort has been followed for many years, many have received information about their PCB and/or PBB levels in the past, and, depending on exposure level or diagnosed health condition, might have been more motiviated to particpate in this study. If both of these factors affected participation, assocations between exposure and health outcomes might be altered. Next, since PCBs have persisted in our environment, results may be confounded due to continued exposure. We did not have lifetime PCB data as our exposure variable was only measured in all participants at the same time as the administration of the health questionnaire^7,46^. Also, the age when first exposed to EDC has been shown to be important for the development of health outcomes^3,11,30,46,53^. However, due to the small number of people exposed *in utero* in this study, this was not able to be analyzed at this time. Additionally, while this population having comparable exposure to PCB as the rest of the U.S population is a strength, there were not many women with high levels of exposure. Therefore, we were not able to evaluate for outcomes at the highest levels of exposure. Another limitation is that multiple comparisons were made in this study which may increase the incidence of Type I errors and can make some of our findings due to chance. Another limitation is that, although majority of outcomes occurred at rates similar to the general population, some were not common, limiting sample size and thus power when evaluating associations. Finally, being in rural Michigan, our study population demographic was 95% White/Non-Hispanic, thus we were unable to evaluate associations by race. However, this is still one of the largest studies looking at multiple reproductive health outcomes in this population.

In conclusion, this study suggests that higher total serum PCB levels were associated with fewer numbers of lifetime pregnancies, but not with other reproductive outcomes. These results build upon previous studies evaluating multiple reproductive health outcomes on exposure to PCB or PBB in women and their children. It is possible that inconsistent results between PCB or PBB exposure and reproductive outcomes is due to the diverse congeners of polyhalogenated organic compounds, unknown confounders, and timing of exposure. Even though PCBs and PBBs are no longer manufactured, exposure to these compounds remain widespread due to their long biologic half-life, accumulation in the food chain, and structurally similar compounds which continue to be produced. Thus, the study of these older chemicals is still relevant and important. Further research and surveillance are needed to determine if and how PCB may decrease pregnancy number.

### Ethics approval and consent to participate

Informed consent was obtained from each individual before participation. Study protocols were approved by the Institutional Review Board at Emory University.

## Supplementary information

Supplementary Tables.

## Data Availability

The datasets used and/or analysed during the current study are available from the corresponding author on reasonable request.

## References

[1] (ATSDR), A. f. T. S. a. D. R. (ed. U.S. Department of Health and Human Services) (ATSDR, Atlanta, GA, 2000).

[2] (ATSDR), A. f. T. S. a. D. R. (ed. U.S. Department of Health and Human Services) (ATSDR, Atlanta, GA, 2004).

[3] Small CM (2007). Risk of spontaneous abortion among women exposed to polybrominated biphenyls. Environmental research.

[4] Gore AC (2015). EDC-2: The Endocrine Society’s Second Scientific Statement on Endocrine-Disrupting Chemicals. Endocrine reviews.

[5] Yang CY (2011). Menstrual effects among women exposed to polychlorinated biphenyls and dibenzofurans. Environmental research.

[6] Buck Louis GM (2011). Persistent organochlorine pollutants and menstrual cycle characteristics. Chemosphere.

[7] Davis SI (2005). Menstrual function among women exposed to polybrominated biphenyls: a follow-up prevalence study. Environmental health: a global access science source.

[8] Cohn BA (2011). Polychlorinated biphenyl (PCB) exposure in mothers and time to pregnancy in daughters. Reproductive toxicology (Elmsford, N.Y.).

[9] Buck Louis GM (2009). Polychlorinated biphenyl serum concentrations, lifestyle and time-to-pregnancy. Human reproduction (Oxford, England).

[10] Chevrier C (2013). Organochlorine pesticides, polychlorinated biphenyls, seafood consumption, and time-to-pregnancy. Epidemiology (Cambridge, Mass.).

[11] Small CM, Murray D, Terrell ML, Marcus M (2011). Reproductive outcomes among women exposed to a brominated flame retardant in utero. Archives of environmental & occupational health.

[12] Han L (2016). In utero exposure to polychlorinated biphenyls is associated with decreased fecundability in daughters of Michigan female fisheaters: a cohort study. Environmental health: a global access science source.

[13] Leoni V (1989). PCB and other organochlorine compounds in blood of women with or without miscarriage: a hypothesis of correlation. Ecotoxicology and environmental safety.

[14] Dar E, Kanarek MS, Anderson HA, Sonzogni WC (1992). Fish consumption and reproductive outcomes in Green Bay, Wisconsin. Environmental research.

[15] Lambertino A, Turyk M, Anderson H, Freels S, Persky V (2011). Uterine leiomyomata in a cohort of Great Lakes sport fish consumers. Environmental research.

[16] Trabert B (2015). Persistent organic pollutants (POPs) and fibroids: results from the ENDO study. Journal of exposure science & environmental epidemiology.

[17] Yang Q (2015). Association of serum levels of typical organic pollutants with polycystic ovary syndrome (PCOS): a case-control study. Human reproduction (Oxford, England).

[18] Yao M (2017). Polychlorinated biphenyls and its potential role in endometriosis. Environmental pollution (Barking, Essex: 1987).

[19] Hoffman CS (2007). Endometriosis among women exposed to polybrominated biphenyls. Annals of epidemiology.

[20] Terrell ML, Hartnett KP, Lim H, Wirth J, Marcus M (2015). Maternal exposure to brominated flame retardants and infant Apgar scores. Chemosphere.

[21] Givens, M. L. *et al*. Maternal exposure to polybrominated and polychlorinated biphenyls: infant birth weight and gestational age. Chemosphere 69, 1295–1304, 10.1016/j.chemosphere.2007.05.031 (2007). 10.1016/j.chemosphere.2007.05.031 PMC2075473 17617441

[22] Lignell S (2013). Prenatal exposure to polychlorinated biphenyls (PCBs) and polybrominated diphenyl ethers (PBDEs) may influence birth weight among infants in a Swedish cohort with background exposure: a cross-sectional study. Environmental health: a global access science source.

[23] Khanjani N, Sim MR (2007). Maternal contamination with PCBs and reproductive outcomes in an Australian population. Journal of exposure science & environmental epidemiology.

[24] Longnecker MP, Klebanoff MA, Brock JW, Guo X (2005). Maternal levels of polychlorinated biphenyls in relation to preterm and small-for-gestational-age birth. Epidemiology (Cambridge, Mass.).

[25] Sweeney, A. M. & Symanski, E. The influence of age at exposure to PBBs on birth outcomes. Environmental research 105, 370–379, 10.1016/j.envres.2007.03.006 (2007). 10.1016/j.envres.2007.03.006 17485077

[26] Terrell, M. L., Small, C. M., Cameron, L. L. & Marcus, M. Comment on “The influence of age at exposure to PBBs on birth outcomes”. Environmental research 108, 117–120; discussion 121–116, doi:10.1016/j.envres.2008.04.007 (2008). 10.1016/j.envres.2008.04.007 PMC2597063 18555987

[27] Casas M (2015). Prenatal exposure to PCB-153, p,p′-DDE and birth outcomes in 9000 mother-child pairs: exposure-response relationship and effect modifiers. Environment international.

[28] Berkowitz GS, Lapinski RH, Wolff MS (1996). The role of DDE and polychlorinated biphenyl levels in preterm birth. Archives of environmental contamination and toxicology.

[29] Ma J, Qiu X, Ren A, Jin L, Zhu T (2012). Using placenta to evaluate the polychlorinated biphenyls (PCBs) and polybrominated diphenyl ethers (PBDEs) exposure of fetus in a region with high prevalence of neural tube defects. Ecotoxicology and environmental safety.

[30] Small CM (2009). Maternal exposure to a brominated flame retardant and genitourinary conditions in male offspring. Environmental health perspectives.

[31] Jaacks LM (2016). Pre-pregnancy maternal exposure to polybrominated and polychlorinated biphenyls and gestational diabetes: a prospective cohort study. Environmental health: a global access science source.

[32] Shapiro GD (2016). Exposure to organophosphorus and organochlorine pesticides, perfluoroalkyl substances, and polychlorinated biphenyls in pregnancy and the association with impaired glucose tolerance and gestational diabetes mellitus: The MIREC Study. Environmental research.

[33] Vafeiadi M (2017). Persistent organic pollutants in early pregnancy and risk of gestational diabetes mellitus. Environ. Int..

[34] Eslami B (2016). Association of serum concentrations of persistent organic pollutants (POPs) and risk of pre-eclampsia: a case-control study. Journal of environmental health science & engineering.

[35] Savitz DA, Klebanoff MA, Wellenius GA, Jensen ET, Longnecker MP (2014). Persistent organochlorines and hypertensive disorders of pregnancy. Environmental research.

[36] Jacobson JL, Fein GG, Jacobson SW, Schwartz PM, Dowler JK (1984). The transfer of polychlorinated biphenyls (PCBs) and polybrominated biphenyls (PBBs) across the human placenta and into maternal milk. American journal of public health.

[37] Fries GF (1985). The PBB episode in Michigan: an overall appraisal. Critical reviews in toxicology.

[38] Joseph AD, Terrell ML, Small CM, Cameron LL, Marcus M (2009). Assessing inter-generational transfer of a brominated flame retardant. Journal of environmental monitoring: JEM.

[39] Landrigan PJ (1979). Cohort study of Michigan residents exposed to polybrominated biphenyls: epidemiologic and immunologic findings. Annals of the New York Academy of Sciences.

[40] Kreiss K (1985). Studies on populations exposed to polychlorinated biphenyls. Environmental health perspectives.

[41] Jacobson MH (2017). Serum Polybrominated Biphenyls (PBBs) and Polychlorinated Biphenyls (PCBs) and Thyroid Function among Michigan Adults Several Decades after the 1973-1974 PBB Contamination of Livestock Feed. Environmental health perspectives.

[42] Safe S (1984). Polychlorinated biphenyls (PCBs) and polybrominated biphenyls (PBBs): biochemistry, toxicology, and mechanism of action. Critical reviews in toxicology.

[43] Hass JR, McConnell EE, Harvan DJ (1978). Chemical and toxicologic evaluation of firemaster BP-6. Journal of agricultural and food chemistry.

[44] Marder ME (2016). Quantification of Polybrominated and Polychlorinated Biphenyls in Human Matrices by Isotope-Dilution Gas Chromatography-Tandem Mass Spectrometry. Journal of analytical toxicology.

[45] Helsel DR (1990). Less than obvious - statistical treatment of data below the detection limit. Environmental science & technology.

[46] Curtis SW (2019). Thyroid hormone levels associate with exposure to polychlorinated biphenyls and polybrominated biphenyls in adults exposed as children. Environ Health.

[47] Curtis, S. W. *et al*. Exposure to polybrominated biphenyl (PBB) associates with genome-wide DNA methylation differences in peripheral blood. Epigenetics, 1–15, doi:10.1080/15592294.2019.1565590 (2019). 10.1080/15592294.2019.1565590 PMC6380401 30676242

[48] Phillips DL (1989). Chlorinated hydrocarbon levels in human serum: effects of fasting and feeding. Archives of environmental contamination and toxicology.

[49] Xue J, Liu SV, Zartarian VG, Geller AM, Schultz BD (2014). Analysis of NHANES measured blood PCBs in the general US population and application of SHEDS model to identify key exposure factors. Journal of exposure science & environmental epidemiology.

[50] CDC. CDC. Fourth National Report on Human Exposure to Environmental Chemicals, https://www.cdc.gov/exposurereport/pdf/fourthreport.pdf (2009). 20806995

[51] Buck Louis GM (2013). Persistent environmental pollutants and couple fecundity: the LIFE study. Environmental health perspectives.

[52] Curtis SW (2018). Intergenerational effects of endocrine-disrupting compounds: a review of the Michigan polybrominated biphenyl registry. Epigenomics.

[53] Small, C. M. *et al*. In utero exposure to a brominated flame retardant and male growth and development. *Int. J. Child and Adolescent Health* **2** (2009). PMC6782048 31595180

[54] Fein GG, Jacobson JL, Jacobson SW, Schwartz PM, Dowler JK (1984). Prenatal exposure to polychlorinated biphenyls: effects on birth size and gestational age. The Journal of pediatrics.

[55] Baibergenova A, Kudyakov R, Zdeb M, Carpenter DO (2003). Low birth weight and residential proximity to PCB-contaminated waste sites. Environmental health perspectives.

[56] Vasiliu O, Cameron L, Gardiner J, Deguire P, Karmaus W (2006). Polybrominated biphenyls, polychlorinated biphenyls, body weight, and incidence of adult-onset diabetes mellitus. Epidemiology (Cambridge, Mass.).

[57] Everett CJ, Mainous AG, Frithsen IL, Player MS, Matheson EM (2008). Association of polychlorinated biphenyls with hypertension in the 1999–2002 National Health and Nutrition Examination Survey. Environmental research.

[58] Goncharov A, Bloom M, Pavuk M, Birman I, Carpenter DO (2010). Blood pressure and hypertension in relation to levels of serum polychlorinated biphenyls in residents of Anniston, Alabama. J. Hypertens.

[59] Hopf NB, Ruder AM, Succop P (2009). Background levels of polychlorinated biphenyls in the U.S. population. The Science of the total environment.

[60] Schecter A (2005). Polybrominated diphenyl ether flame retardants in the U.S. population: current levels, temporal trends, and comparison with dioxins, dibenzofurans, and polychlorinated biphenyls. Journal of occupational and environmental medicine.

[61] Sjodin A (2004). Retrospective time-trend study of polybrominated diphenyl ether and polybrominated and polychlorinated biphenyl levels in human serum from the United States. Environmental health perspectives.

