# Supplementary material for: Examining Reproductive Health Outcomes in Females Exposed to Polychlorinated Biphenyl and Polybrominated Biphenyl
Source: Sci Rep. 2020 Feb 24;10:3314. doi: 10.1038/s41598-020-60234-9 (PMC7039953; doi:10.1038/s41598-020-60234-9)
Supplement: Supplementary file 1 — Supplementary Tables. [file 41598_2020_60234_MOESM1_ESM.docx]

**Title:**

**Examining Reproductive Health Outcomes in Females Exposed to Polychlorinated Biphenyl and Polybrominated Biphenyl**

**Authors:**

1. Michael F. Neblett II, Department of Gynecology and Obstetrics, Emory University School of Medicine, 101 Woodruff Circle NE, Ste 4300, Atlanta, GA 30322, [mfneble@emory.edu](mailto:mfneble@emory.edu)

2. Sarah W. Curtis, Genetics and Molecular Biology Program, Laney Graduate School, Emory University School of Medicine, 101 Woodruff Circle NE, Ste 2205A, Atlanta, GA 30322, [sarah.elizabeth.whelan@emory.edu](mailto:sarah.elizabeth.whelan@emory.edu)

3. Sabrina A. Gerkowicz, Department of Gynecology and Obstetrics, Emory University School of Medicine, 101 Woodruff Circle NE, Ste 4300, Atlanta, GA 30322, [sgerkov@emory.edu](mailto:sgerkov@emory.edu)

4. Jessica B. Spencer, Department of Gynecology and Obstetrics, Emory University School of Medicine, 101 Woodruff Circle NE, Ste 4300, Atlanta, GA 30322, [jbspenc@emory.edu](mailto:jbspenc@emory.edu)

5. Metrecia L. Terrell, Department of Epidemiology, Emory University Rollins School of Public Health, 1518 Clifton Rd, Atlanta, GA 30322, [mterrel@emory.edu](mailto:mterrel@emory.edu)

6. Victoria S. Jiang, Department of Gynecology and Obstetrics, Emory University School of Medicine, 101 Woodruff Circle NE, Ste 4300, Atlanta, GA 30322, [victoria.shuangbai.jiang@emory.edu](mailto:victoria.shuangbai.jiang@emory.edu)

7. M. Elizabeth Marder, Department of Environmental Health, Emory University Rollins School of Public Health, 1518 Clifton Rd, Atlanta, GA 30322, [m.elizabeth.marder@gmail.com](mailto:m.elizabeth.marder@gmail.com)

8. Dana Boyd Barr, Department of Environmental Health, Emory University Rollins School of Public Health, 1518 Clifton Rd, Atlanta, GA 30322, [dbbarr@emory.edu](mailto:dbbarr@emory.edu)

9. Michele Marcus, Department of Epidemiology, Rollins School of Public Health; Department of Environmental Health, Rollins School of Public Health; Department of Pediatrics, Emory University School of Medicine, 1518 Clifton Rd, Atlanta, GA 30322, [mmarcus@emory.edu](mailto:mmarcus@emory.edu)

10. Alicia K. Smith, Department of Gynecology and Obstetrics, Emory University School of Medicine, 101 Woodruff Circle NE, Ste 4217, Atlanta, GA 30322, [alicia.smith@emory.edu](mailto:alicia.smith@emory.edu)

**Corresponding Author:**

Michael F. Neblett II

Emory University School of Medicine

Glenn Building, 4^th^ Floor – 412 B

69 Jesse Hill Jr. Drive SE

Atlanta, GA 30303

[mfneble@emory.edu](mailto:mfneble@emory.edu)

**Supplementary Tables:**

**Table S1: Sensitivity Analyses**

|  | N (%) | **PCB** | | **PBB** | |
| --- | --- | --- | --- | --- | --- |
| **Infertility Risk Factors^a^** |  | OR (95% CI) | P-value | OR (95% CI) | P-value |
| Any report of going 6+ months with regular unprotected intercourse and DID NOT achieve pregnancy? | 44 (18.8%) | 1.40 (0.96 to 2.08) | 0.09 | 0.99 (0.70 to 1.40) | 0.96 |
| Any report of going 12+ months with regular unprotected intercourse and DID NOT achieve pregnancy? | 26 (11.1%) | 1.23 (0.76 to 2.01) | 0.40 | 1.12 (0.72 to 1.74) | 0.62 |
| Any report of going 6+ months with regular unprotected intercourse without becoming pregnant? | 119 (50.8%) | 1.24 (0.92 to 1.68) | 0.17 | 0.91 (0.70 to 1.18) | 0.47 |
| Any report of going 12+ months with regular unprotected intercourse without becoming pregnant? | 77 (32.9%) | 1.05 (0.75 to 1.46) | 0.79 | 0.89 (0.67 to 1.18) | 0.44 |
| Have you ever had a problem or been concerned about possible problem with ability to get pregnant? | 70 (29.9%) | 0.93 (0.66 to 1.31) | 0.69 | 1.07 (0.80 to 1.43) | 0.62 |
| Have you ever visited a health care provider, doctor, or clinic because you were having difficulty getting pregnant? | 43 (18.3%) | 0.79 (0.51 to 1.20) | 0.27 | 0.98 (0.68 to 1.39) | 0.91 |
| Reports receiving fertility treatments | 29 (12.3%) | 0.87 (0.53 to 1.42) | 0.58 | 0.85 (0.56 to 1.29) | 0.46 |
|  |  |  |  |  |  |
| **Infertility Risk Factors^a^** | Mean (Range) | $\beta$ (95% CI) | P-value | $\beta$ (95% CI) | P-value |
| Number of pregnancies | 2.84 (0-10) | **-0.11 (-0.21 to -0.005)** | **0.03** | 0.02 (-0.06 to 0.09) | 0.66 |
|  |  |  |  |  |  |
| **Miscarriage Risk Factors^b^** | N (%) | OR (95% CI) | P-value | OR (95% CI) | P-value |
| Miscarriage | 80 (17.8%) | 1.14 (0.84 to 1.54) | 0.38 | 0.94 (0.75 to 1.17) | 0.59 |
|  |  |  |  |  |  |
| **Preterm Birth Risk Factors^b^** | N (%) | OR (95% CI) | P-value | OR (95% CI) | P-value |
| Preterm Birth^d^ | 35 (9.5%) | 0.94 (0.59 to 1.51) | 0.82 | 0.99 (0.74 to 1.35) | 0.99 |
|  |  |  |  |  |  |
| **Birth Weight Risk Factors^c^** | N (%) | OR (95% CI) | P-value | OR (95% CI) | P-value |
| Low birth weight^e^ | 23 (6.2%) | 1.01 (0.61 to 1.67) | 0.96 | 1.11 (0.77 to 1.61) | 0.56 |
| High birth weight^f^ | 39 (10.6%) | 0.69 (0.43 to 1.12) | 0.13 | 0.95 (0.70 to 1.30) | 0.78 |
|  | Mean (Range) | $\beta$ (95% CI) | P-value | $\beta$ (95% CI) | P-value |
| Birth weight (grams) | 3351g (1219-5415g) | -63.1 (-157.9 to 31.7) | 0.19 | 32.1 (-27.3 to 91.6) | 0.29 |

Adjusted for age, BMI, and total lipid levels. Total PCB levels adjusted for total PBB and total PBB levels adjusted for total PCB. OR= odds ratio. CI= confidence interval.

Bold indicates statistically significant values with p-value of <0.05.

^a^ Additionally adjusted for endometriosis, polycystic ovarian syndrome, uterine fibroids, and sexually transmitted infections with link to infertility.

^b^ Additionally adjusted for polycystic ovarian syndrome, uterine fibroids, hypertensive disorders of pregnancy, and diabetes in pregnancy.

^c^ Additionally adjusted for hypertensive disorders of pregnancy and diabetes in pregnancy.

^d^ <37 weeks gestational age

^e^ Self-reported birth weight <2500g

^f^ Self-reported birth weight >4000g

**Table S2: Associations between exposures and gynecological outcomes not adjusted for total lipid levels**

|  | N (%) | **PCB** | | **PBB** | | |
| --- | --- | --- | --- | --- | --- | --- |
|  |  | OR (95% CI) | P-value | OR (95% CI) | P-value |  |
| **PID^a^** | 13 (5.1%) | 1.42 (0.74 to 2.90) | 0.30 | 1.09 (0.62 to 1.85) | 0.74 |  |
| **Endometriosis** | 44 (17.3%) | 1.01 (0.68 to 1.53) | 0.93 | 1.04 (0.75 to 1.43) | 0.79 |  |
| **PCOS^b^** | 23 (9.0%) | 0.96 (0.57 to 1.60) | 0.87 | 1.02 (0.64 to 1.57) | 0.91 |  |
| **Uterine Fibroids** | 20 (7.8%) | 0.70 (0.37 to 1.30) | 0.27 | 0.90 (0.56 to 1.42) | 0.66 |  |

Adjusted for age and BMI. Total PCB levels adjusted for total PBB and total PBB levels adjusted for total PCB. OR= odds ratio. CI= confidence interval.

^a^ Pelvic inflammatory disease

^b^ Polycystic ovarian syndrome

|  | N (%) | **PCB** | | **PBB** | |
| --- | --- | --- | --- | --- | --- |
|  |  | OR (95% CI) | P-value | OR (95% CI) | P-value |
| Any report of going 6+ months with regular unprotected intercourse and DID NOT achieve pregnancy? | 44 (18.8%) | 1.42 (0.98 to 2.09) | 0.06 | 1.00 (0.71 to 1.38) | 0.98 |
| Any report of going 12+ months with regular unprotected intercourse and DID NOT achieve pregnancy? | 26 (11.1%) | 1.28 (0.81 to 2.07) | 0.28 | 1.11 (0.74 to 1.65) | 0.59 |
| Any report of going 6+ months with regular unprotected intercourse in life without becoming pregnant? | 119 (50.8%) | 1.23 (0.91 to 1.67) | 0.16 | 0.90 (0.70 to 1.16) | 0.45 |
| Any report of going 12+ months with regular unprotected intercourse in life without becoming pregnant? | 77 (32.9%) | 1.04 (0.75 to 1.43) | 0.80 | 0.88 (0.67 to 1.16) | 0.39 |
| Have you ever had a problem or been concerned about possible problem with ability to get pregnant? | 70 (29.9%) | 0.93 (0.67 to 1.29) | 0.66 | 1.03 (0.78 to 1.36) | 0.79 |
| Have you ever visited a health care provider, doctor, or clinic because you were having difficulty getting pregnant? | 43 (18.3%) | 0.77 (0.52 to 1.15) | 0.21 | 0.90 (0.64 to 1.24) | 0.54 |
| Reports receiving fertility treatments | 29 (12.3%) | 0.84 (0.53 to 1.33) | 0.46 | 0.81 (0.54 to 1.18) | 0.28 |

**Table S3: Association between exposures and infertility outcomes not adjusted for total lipid level**

Adjusted for age and BMI. Total PCB levels adjusted for total PBB and total PBB levels adjusted for total PCB. OR= odds ratio. CI= confidence interval.

**Table S4: Associations between exposures and pregnancy outcomes not adjusted for total lipid level**

|  | Mean (range) | **PCB** | | **PBB** | |
| --- | --- | --- | --- | --- | --- |
|  |  | $\beta$ (95% CI) | P-value | $\beta$ (95% CI) | P-value |
| **Number of pregnancies** | 2.84 (0-10) | **-0.11 (-0.21 to -0.003)** | **0.04** | 0.02 (-0.06 to 0.09) | 0.60 |
|  | | | | | |
| **Pregnancy Outcomes** | N (%) | OR (95% CI) | P-value | OR (95% CI) | P-value |
| Singleton live birth (SLB) | 369 (82.2%) | 0.88 (0.64 to 1.21) | 0.44 | 1.02 (0.83 to 1.24) | 0.86 |
| Miscarriage | 80 (17.8%) | 1.13 (0.82 to 1.55) | 0.44 | 0.98 (0.80 to 1.20) | 0.86 |
|  | | | | | |
| **SLB Outcomes** | N (%) | OR (95% CI) | P-value | OR (95% CI) | P-value |
| Preterm birth^a^ | 35 (9.5%) | 0.97 (0.61 to 1.52) | 0.89 | 0.97 (0.71 to 1.34) | 0.87 |
| Low birth weight^b^ | 23 (6.2%) | 1.02 (0.61 to 1.69) | 0.93 | 1.10 (0.74 to 1.61) | 0.64 |
| High birth weight^c^ | 39 (10.6%) | 0.68 (0.43 to 1.09) | 0.11 | 0.94 (0.69 to 1.30) | 0.72 |
| Birth defects | 26 (7.0%) | 0.97 (0.57 to 1.66) | 0.92 | 0.93 (0.63 to 1.39) | 0.75 |
| Hypertensive disorders of pregnancy^d^ | 29 (7.9%) | 1.54 (0.90 to 2.61) | 0.11 | 1.32 (0.86 to 2.00) | 0.19 |
| Gestational diabetes | 25 (6.8%) | 1.08 (0.57 to 2.02) | 0.81 | 1.04 (0.59 to 1.84) | 0.88 |
|  | | | | | |
|  | Mean (range) | $\beta$ (95% CI) | P-value | $\beta$ (95% CI) | P-value |
| **Birth weight (grams)** | 3351g (1219-5415g) | -63.7 (-159.3 to 31.9) | 0.19 | 32.8 (-29.2 to 94.9) | 0.30 |

Adjusted for age (current age & age at pregnancy) and BMI. Total PCB levels adjusted for total PBB and total PBB levels adjusted for total PCB. OR= odds ratio. CI= confidence interval.

Bold indicates statistically significant values with p-value of <0.05.

^a^ <37 weeks gestational age

^b^ Self-reported birth weight <2500g

^c^ Self-reported birth weight >4000g

^d^ Gestational hypertension, preeclampsia, or eclampsia
